# “I just thought I was lucky to be protected from HIV:” Qualitative evaluation of barriers and facilitators of pre-exposure prophylaxis use for adolescent girls and young women at higher risk of HIV acquisition in Lilongwe, Malawi

**DOI:** 10.1371/journal.pone.0332643

**Published:** 2025-10-27

**Authors:** Mallory Michalak, Odala Sande, Sam Phiri, Hannock Tweya, Geldert Chiwaya, Joseph Chintedza, Beatrice Matanje, Christine Kiruthu-Kamamia, Jacqueline Huwa, Jane Chiwoko, Tom Chaweza, Rose Nyirenda, Thokozani Kalua, Andreas Jahn, Alinafe Mbewe, Alice Maida, Evelyn Kim, Wezi Msungama, Fatima Zulu, Mtemwa Nyangulu, Sarah Shaw, Victor Singano, Newton Kalata, Alinune Kabaghe, Pragna Patel

**Affiliations:** 1 United States Centers for Disease Control and Prevention, Division of Global HIV and Tuberculosis, Atlanta, Georgia, United States of America; 2 United States Centers for Disease Control and Prevention, Division of Global HIV and Tuberculosis, Lilongwe, Malawi; 3 Lighthouse Trust, Lilongwe, Malawi; 4 Ministry of Health, Lilongwe, Malawi; 5 RWD Consulting, LLC, Seattle, Washington, United States of America; Public Library of Science, UNITED KINGDOM OF GREAT BRITAIN AND NORTHERN IRELAND

## Abstract

Adolescent girls and young women (AGYW) are at higher risk of HIV acquisition than their male counterparts, especially in sub-Saharan African countries such as Malawi. Therefore, HIV prevention programming is a key component to reducing this risk. We conducted a formative qualitative assessment with AGYW and their health providers to understand AGYW’s perceptions of their own HIV risk, self-efficacy to protect themselves, and the implications of these factors on pre-exposure prophylaxis (PrEP) use. Our study found that AGYW are aware and interested in using PrEP, which can allow choices that protect their sexual health and well-being. Innovative service delivery models that minimize stigma and offer other reproductive health services such as contraception are needed to provide comprehensive care. Additionally, AGYW and their health providers would benefit from education about HIV risk perception and PrEP effectiveness to improve use of PrEP. Robust AGYW PrEP service delivery, including new biomedical prevention strategies, may facilitate efforts to achieve epidemic control.

## Introduction

Adolescent girls and young women (AGYW) account for a disproportionate amount of new HIV infections throughout southern Africa. From 2000 to 2017, approximately 38–63% of new HIV infections in southern and eastern Africa occurred among AGYW aged 15–24 years [1]. In Malawi, AGYW aged 15–24 years are more than twice as likely to be living with HIV than their male peers [2] and have the highest annual incidence of HIV in the country among all age and sex demographic groups [3]. Many of the risk factors associated with HIV acquisition for young women in the region [4], including sexually transmitted infections (STIs) and intergenerational sexual relationships, have been found to be predictive of HIV incidence among AGYW in Malawi [5].

Given the importance of preventing HIV infection among AGYW in order to reach and sustain HIV epidemic control [6], this population should be prioritized for pre-exposure prophylaxis (PrEP) [7]. In 2020, the Malawian Ministry of Health (MoH) released the national policy approving the use of PrEP for populations disproportionately affected by HIV, including AGYW, female sex workers, and HIV sero-different couples [8]. However, there are myriad challenges associated with the uptake, adherence, and scale-up of PrEP. Basic knowledge of PrEP among health care providers and potential users, particularly among AGYW and other populations at risk for HIV, remains low [9,10] and retention is a notable programmatic challenge [11]. Furthermore, research suggests that early demand for PrEP among AGYW in Malawi may not be well aligned with epidemiologic risk and that further efforts are needed to improve engagement with PrEP among this target population [12]. This study aims to build on the body of research exploring health providers’ perspectives on providing PrEP to AGYW in the region [13,14] and on research exploring PrEP interest and service delivery preferences among populations with high risk for HIV acquisition in Malawi [10,15,16].

Therefore, we conducted a formative qualitative assessment with AGYW and PrEP health care providers from two health facilities in urban areas within the capital city of Lilongwe, Malawi during a PrEP demonstration project to understand barriers and facilitators of PrEP use among AGYW. This study sought to identify AGYW’s perception of their own risk of HIV acquisition, the factors that influence their decision to begin and continue a daily PrEP regimen, and preferences for service delivery.

## Methodology

### Study design and setting

This qualitative formative assessment was conducted from January to June of 2020 as part of a mixed-methods prospective cohort study. The qualitative work was conducted in the six months immediately following study initiation and was aimed at assessing the feasibility of PrEP delivery as an HIV prevention strategy for AGYW. Bwaila District Hospital and Kawale Health Center were selected based on the facilities’ HIV burden and site appropriateness for the study objectives: Lilongwe has an HIV prevalence of 9.0% among people aged 15–49 years [3] and both study sites are high-volume facilities for the provision of antiretroviral therapy, STI diagnosis and treatment, and reproductive health services.

### Ethical approval and informed consent

This study was reviewed and approved by the Malawi National Health Sciences Research Committee (NHSRC approval #1994) and the U.S. Centers for Disease Control and Prevention (CDC). Study procedures were explained to eligible participants and all participants provided written consent.

### Inclusivity in global research

Additional information regarding the ethical, cultural, and scientific considerations specific to inclusivity in global research is included in the Supporting Information (S1 Checklist).

### Participant recruitment and eligibility

AGYW aged 18–24 years who met the following criteria were invited to enroll in the mixed-methods study: (1) had an ongoing risk of HIV acquisition (operationally defined as being sexually active within the last 6 months), (2) had an unknown HIV status or had not had an HIV test within the past 3 months, and (3) were eligible for PrEP, as determined by the eligibility criteria [17] described below in Table 1. Additional criteria included residing in either of the health facility catchment areas and not planning to relocate within the next 12 months from time of recruitment. AGYW were recruited through referrals from youth-friendly health centers and community outreach activities.

**Table 1:** Eligibility criteria for PrEP study and qualitative study participation: Malawi, 2020.

Sex	Female
Age	18-24 years
HIV status	HIV negative and have no symptoms of acute HIV infection
Weight	>35 kg
Breastfeeding status	Not currently breastfeeding
Tuberculosis	Not currently receiving treatment for multi-drug resistant tuberculosis
Liver disease	No history of liver or renal disease
Study enrollment	Not currently enrolled in another study providing PrEP (either oral or injectable)
Substantial HIV risk (Must meet at least 2 criteria)	1) had a new sexual partner in the last 3 months 2) had more than one sexual partner in the last 3 months 3) has an STI or had an STI in the last 6 months 4) formally and regularly exchanges sexual services in return for cash or payment in kind

AGYW who were enrolled in the mixed-method study were invited to participate in the qualitative assessment, which utilized methods triangulation [18] to incorporate both focus group discussions (FGDs) and in-depth interviews (IDIs). Beyond participation in FGDs and IDIs, all participants received quarterly follow up: those who initiated PrEP received quarterly HIV testing and medication dispensing; those who declined PrEP received quarterly HIV testing and counseling. All AGYW participants were also offered information, education, and communication materials on the following topics: HIV prevention strategies, PrEP use and effectiveness, and services for intimate partner violence. FGDs were conducted with providers trained to administer PrEP at both facilities.

All AGYW who participated in FGDs and IDIs received a stipend to cover the cost of transportation (3,000 Malawian kwacha/approximately 4USD at the time recruitment) to the study site for each visit.

Health care providers who met the following criteria were eligible for participation in the formative assessment’s FGDs: (1) were a trained nurse or clinician employed at either of the two health facilities participating in the study, (2) had provided PrEP for at least 3 months, and (3) were trained in the procedures of the PrEP study, including AGYW participant enrollment and PrEP service provision.

### Sampling

For FGDs among AGYW, purposeful sampling was used for participant selection and grouped by the following demographics: marital status (married [n = 18]; unmarried [n = 43]) and PrEP use (accepted [n = 53]; declined [n = 8]). For IDIs among AGYW, purposeful sampling was used for participant selection in IDIs according to PrEP status: accepted and currently taking PrEP (n = 5); eligible for but did not accept PrEP (n = 5); accepted but later discontinued PrEP (n = 5). PrEP status was selected as criterion to understand barriers to and facilitators of PrEP use from both populations; marital status was selected based on existing research on HIV risk profiles among AGYW in the region [5,19,20].

For FGDs among health care providers, two were conducted with all health care providers engaged in the study and were grouped according to cadre: nurses (n = 3) and clinicians (n = 4). Due to the demands on health care providers’ time, recruitment of only a small sample was possible, and FGDs were utilized as a more efficient method of data collection to understand collective experiences, rather than IDIs.

### Discussion guides and data collection

Protective Motivation Theory (PMT) [21] was used as a framework to develop the FGD and IDI guides (see Table 2 below), focusing on the cognitive mediating processes of threat appraisal and coping appraisal. This theory was selected as a model because it allows examination of various themes of interest among AGYW and the health providers who serve them, including developmentally relevant variables (e.g., adolescent perceptions of invulnerability and changing relationships with parents and peers), as well as potentially relevant economic, historical, and cultural influences in the community [22]. Furthermore, this theory has been used to assess sexual risk reduction behaviors among adolescents in other contexts [23,24] and to guide the development of various health behavior interventions [25,26].

**Table 2:** Protection Motivation Theory in Data Collection for PrEP qualitative study: Malawi, 2020.

Domain	Subdomain	FGD and IDI questions
Threat appraisal	Severity	How does HIV/AIDS affect adolescent girls and young women?
	Vulnerability	What do you think are factors that put young women at risk for getting HIV/AIDS?
Coping appraisal	Response effectiveness	What services or methods are available for young women to prevent HIV?
	Response costs	What are the barriers to using these HIV preventive measures?

The FGD and IDI guides were developed in English, translated into the local language (Chichewa), then back-translated into English to ensure accuracy [27]. The guides included the following topics: awareness of HIV risk factors; barriers to HIV testing among AGYW; perception of HIV/AIDS burden and risk among AGYW; characteristics of AGYW’s social networks and sexual behavior of their friends; acceptability and feasibility of PrEP for high-risk AGYW; and barriers to adherence and retention on PrEP.

Each FGD and IDI was facilitated by a trained facilitator/interviewer who spoke Chichewa and was familiar with local cultural norms and experiences to better understand the context of participant responses [28]. AGYW and health care provider FGDs were conducted in Chichewa and were audio recorded. FGDs with providers lasted between 45 minutes and one hour; FGDs with AGYW lasted between 90 minutes and two hours. Seven FGDs were conducted with AGYW; each group consisted of 8–10 participants. Fifteen IDIs were conducted in Chichewa with AGYW. IDIs lasted between 40 and 90 minutes. Demographic characteristics were collected for all FGD and IDI participants, including age, PrEP use, employment, education, and marital status among AGYW, as well as sex, age, and years of service among providers. All FGD/IDI data were de-identified and basic demographic information was linked to participation in the qualitative work using a unique and confidential identifier.

### Data analysis

The goal of this analysis was to better understand AGYW’s perceptions of their own HIV risk, self-efficacy to protect themselves, and the implications of these factors on PrEP use. This analysis used a hybrid methodological approach with both inductive and deductive coding; deductive coding was grounded in PMT. We conducted a thematic analysis and generated themes within the overarching process outlined in PMT (threat appraisal versus coping appraisal, protection motivation, and sources of information), as well as decision-making influences, general HIV information, perceptions of PrEP, PrEP delivery, and stigma related to both HIV and PrEP.

All FGDs and IDIs were transcribed and then translated into English by a bilingual (English/Chichewa) researcher. Using analyst triangulation [18], a team of three researchers developed a codebook using a combination of theory-driven codes derived from PMT, such as vulnerability and self-efficacy, and *a priori* provisional/structural codes [29] informed by the interview guide. The research team also utilized peer debriefing [30] to present preliminary findings and solicit feedback on data interpretation. The finalized codes were used to generate themes according to the formative assessment’s objectives: to identify AGYW’s perception of their own risk of HIV acquisition, the factors that influence their decision to begin and continue a daily PrEP regimen, and preferences for service delivery. These themes are organized as subheadings in the Results section.

All transcripts were coded by three different researchers using ATLAS.ti v9. The team organized regular meetings to discuss discrepancies, reach consensus, and redefine codebook definitions as necessary. Following the completion of consensus coding [31], coders independently reviewed the additional transcripts and conducted a review of each transcript to discuss areas of disagreement to increase results reliability [32].

Additionally, Chi square tests were conducted using Stata v18.0 to determine if there was a statistically significant difference between AGYW who initiated PrEP and those who declined based on the following demographic variables: marital status, level of education completed, and self-report of transactional sex within the past 6 months.

## Results

A total of 76 AGYW, with a median age of 20 years (IQR 19–23), participated in the formative qualitative assessments; 61 AGYW participated in FGDs and 15 AGYW participated in IDIs. Of the total sample of AGYW, 63 (83%) elected to take PrEP. The majority (85%) were unemployed and 48% had completed upper secondary education. Among participants, 58% were single, 33% were married, and 9% were divorced/separated. There were no significant differences in PrEP uptake according to level of education or marital status.

Additionally, 58% of AGYW participants reported having multiple sexual partners within the past 3 months and 26% reported having experienced intimate partner violence. Only 3% reported having used condoms in all sexual encounters in the past 6 months and 39% reported having exchanged sex for favors or material support within the past 6 months. Furthermore, there was a statistically significant difference in PrEP uptake among AGYW who reported transactional sex versus those who did not: *X*^*2*^ (1, *N* = 76) = 10.23, p < .001; all AGYW who reported transactional sex within the past 6 months elected to take PrEP. Table 3 also presents demographic results for those who participated in FGDs and IDIs, stratified by data collection type.

**Table 3:** Demographic characteristics for AGYW FGD and IDI participants at enrollment: Malawi, 2020.

Characteristics	FGD (N = 61)	IDI (N = 15)	Total (N = 76)
Median age (IQR)*	20 (19-23)	20 (18-22)	20 (19-23)
Marital status			
Single	39 (64%)	5 (33%)	44 (58%)
Married	18 (30%)	7 (47%)	25 (33%)
Divorced/separated	4 (6%)	3 (20%)	7 (9%)
Highest level of education completed			
Primary	7 (12%)	1 (7%)	8 (11%)
Lower Secondary	16 (26%)	10 (66%)	26 (34%)
Upper Secondary	34 (56%)	3 (20%)	37 (48%)
Tertiary	4 (6%)	1 (7%)	5 (7%)
Employment status			
Unemployed	55 (90%)	10 (67%)	65 (85%)
Employed	6 (10%)	5 (33%)	11 (15%)
Most recent HIV test			
Never	11 (18%)	1 (7%)	12 (16%)
≤3 months ago	33 (54%)	8 (53%)	41 (54%)
>3 months ago	17 (28%)	6 (40%)	23 (30%)
Used condoms in all sexual encounters in the past 6 months			
No	60 (98%)	14 (93%)	74 (97%)
Yes	1 (2%)	1 (7%)	2 (3%)
Screened positive for IPV*			
No	46 (75%)	10 (67%)	56 (74%)
Yes	15 (25%)	5 (33%)	20 (26%)
New sexual partner within past 3 months			
No	38 (62%)	12 (80%)	50 (66%)
Yes	23 (38%)	3 (20%)	26 (34%)
Multiple sexual partners within past 3 months			
No	22 (36%)	10 (67%)	32 (42%)
Yes	39 (64%)	5 (33%)	44 (58%)
Ever exchanged sex for favors or material support			
No	36 (59%)	12 (80%)	48 (63%)
Yes	25 (41%)	3 (20%)	28 (37%)
Exchanged sex for favors or material support within the past 6 months			
No	33 (54%)	13 (87%)	46 (61%)
Yes	28 (46%)	2 (13%)	30 (39%)
Had STI* within the past 6 months			
No	54 (89%)	11 (73%)	65 (85%)
Yes	7 (11%)	4 (27%)	11 (15%)
Had STI within the past 3 months			
No	54 (89%)	13 (87%)	67 (88%)
Yes	7 (11%)	2 (13%)	9 (12%)
Has/had sexual partner who is HIV positive within the past 6 months			
Don’t know	39 (64%)	1 (7%)	40 (53%)
No	20 (33%)	13 (86%)	33 (43%)
Yes	2 (3%)	1 (7%)	3 (4%)

*: IQR=Interquartile range IPV=Intimate partner violence STI=Sexually transmitted infection.

Seven health care providers participated in the FGDs, of which five were women, two men; four were clinicians and three were nurses. The median age of the health care providers was 36 years (IQR 36–37) and they had an average of 11.6 years of service.

### Perception of severity and stigma of an HIV diagnosis

Many AGYW participants spoke to the severity of an HIV diagnosis, particularly as it marks the starting point for managing a chronic condition: *“When one has been diagnosed with HIV you actually know that it is lifelong disease, you will be taking the drugs for the rest of your life, unlike other health problems where you get sick and get well within two or three days”* (AGYW, FGD, unmarried, accepted PrEP). AGYW also spoke directly to the burden of HIV stigma: *“You think that your life is over [with an HIV diagnosis] and you cannot achieve anything”* (AGYW, IDI, accepted and taking PrEP). Participants shared that AGYW living with HIV may encounter difficulties making or socializing with friends if their HIV status is known: *“It affects her in such a way that she will not be able to socialize with her friends especially in cases where her status is known to her friends. The friends may judge her”* (AGYW, IDI, accepted but later discontinued PrEP). Furthermore, the weight of this stigma bears down upon other relationships and life milestones: *“The moment people know that you are HIV positive, they say a lot of things and you are discriminated”* (AGYW, FGD, unmarried, accepted PrEP; *“It may take a long time for one to find a marriage partner”* (AGYW, FGD, unmarried, declined PrEP).

### Perception of AGYW’s vulnerability and stigma related to HIV

Health care providers and AGYW were aware of the multiple HIV-related risk factors faced by AGYW. Poverty and financial instability were cited as root causes for many of these risk factors, with one provider noting *“[Study participants] lack a lot of things. They do not have financial support since they live with a single parent or guardian, whilst some live alone. I used to ask some of them at enrollment as to why they indulge in certain behavior, and they said that it is because their parents cannot manage to support them financially. As such, they resolve to finding a man so that he can be able to give them money so that they can support themselves”* (clinician, FGD).

Lack of financial stability contributed to AGYW’s inability to negotiate condom use, thereby increasing vulnerability to HIV. For AGYW engaged in commercial sex, clients often pay more for sex acts without a condom: *“… they can give you 60,000 [Malawian kwacha, approximately 80USD] and tell you ‘Let’s not use condoms,’ you cannot refuse”* (AGYW, FGD, married, accepted PrEP)*; “… maybe 20,000 or 10,000 or 5,000 [with a condom] because she did not accept what he wants”* (AGYW, FGD, married, accepted PrEP). For AGYW with intimate partners, the desire to use a condom may be outweighed by financial manipulation: *“[When] one has a partner who provides for everything, and that partner has told her to have sex and then you suggest that he uses the condom, he refuses. He tells you that ‘If you want me to use the condom, then that is the end of us.’ In this case the girl accepts to have unprotected sex”* (AGYW, FGD, unmarried, accepted PrEP).

Given these risk factors, AGYW participants often spoke to their own susceptibility to HIV: *“We realized that he is HIV positive at the time that we were getting back together, to say the truth, I am scared of the disease”* (AGYW, FGD, married, accepted PrEP). Many participants voiced that norms and expectations around sex and relationships made it difficult for them to mitigate behaviors and situations that they knew put them at risk for HIV: *“… a woman cannot tell a man to get tested [for HIV], it will be disrespectful. You can use his friends to tell him to get tested, he may not do it right away but will at some point know that his friend was right and may get tested. Men find it difficult to be told what to do by a woman”* (AGYW, FGD, married, accepted PrEP).

### Autonomy and protection from specific risk factors: AGYW’s motivation for PrEP use

Given AGYW’s perception of HIV risk and their own vulnerability, their motivation to use PrEP is often tied to risk factors outside of their locus of control, such as not knowing their partner’s HIV status or their partner’s behavior outside of the relationship. One participant indicated that taking PrEP was preferable to being unsure about her partner’s HIV status: *“In my case whenever I ask my boyfriend to get tested… he tells me that ‘If you’re okay, then I am also fine.’ I decided to be taking PrEP because of that response. He always says that if I am negative, then he is also negative”* (AGYW, FGD, married, accepted PrEP Participants noted that PrEP gave them peace of mind in situations when they would otherwise be unable to reduce their risk: *“We used to be anxious about our partners’ behavior, but we cannot control that. Nowadays we are no longer worried; they can behave however they like, as long as we are taking PrEP”* (AGYW, FGD, married accepted PrEP,). These quotes demonstrate AGYW’s self-efficacy in coping with the threat of HIV, and subsequent intention to reduce their personal risk by using PrEP.

Participants also noted challenges with regards to HIV prevention strategies that require a partner’s consent, specifically condoms. Many participants noted that their partners do not allow consistent condom usage: *“I still use [condoms] when my boyfriend agrees… unless when he refuses”* (AGYW, FGD, married, accepted PrEP). Other participants noted that the request to use a condom would result in a partner stating *“that you do not trust them and have to terminate the relationship”* (AGYW, FGD, unmarried, accepted PrEP). As such, an HIV prevention method that could be used autonomously, such as PrEP, was viewed favorably given its lower response cost (i.e., fewer barriers to use, as partner consensus isn’t required.)

### Perceptions of PrEP use: Varied views

AGYW participants described negative perceptions of a person who uses PrEP within their communities, stating that others may think “*that you are involved in bad behavior and sleeping with different people and that explains why you are taking the drugs”* (AGYW, FGD, married, accepted PrEP,). Other participants noted that *“I think what can discourage a girl from taking PrEP is the way people talk, people say a lot, that one sleeps around and have unprotected sex, so in fear of those insults one may decide to stop taking PrEP”* (AGYW, FGD, unmarried, accepted PrEP). AGYW also noted negative reactions if their sexual partners were to take PrEP: *“I would say we are being deceived; those are ARVs* [*antiretrovirals for HIV treatment]”* (AGYW, FGD, married, accepted PrEP,). Despite having received PrEP education, AGYW were still sensitive to the similarities between PrEP and ARVs, as well as the implications and stigma of a sexual partner with HIV.

Despite these perceptions, AGYW frequently described PrEP as being positive and empowering for themselves, regardless of others’ views: *“As for me I have accepted my fate and I do this wisely, I protect myself. I cannot be following what one is doing, I must do what will help me”* (AGYW, FGD, unmarried, accepted PrEP). Even in the context of stigma around sex and HIV, AGYW had an overall positive perception of PrEP as a means of exercising their own agency and the ability to protect themselves. These nuanced views demonstrate the complexity of AGYW’s cognitive mediating processes in deciding if and how to modify their behavior in order to prevent HIV.

### Barriers to adherence: PrEP literacy and stigma

A common theme among health care providers was a concern regarding AGYW’s adherence to a daily PrEP regimen. Many providers reported that AGYW would only take PrEP when they were about to have sex or on days they were likely to be sexually active: *“Due to maybe poor understanding some would actually say that they have not been taking the drugs for one month as they were at their home village and didn’t see the need to be taking the drugs because their sexual partner was not around at the time”* (clinician, FGD). AGYW participants also noted this misunderstanding, with one participant saying that she stopped taking PrEP upon giving birth: *“I just accepted because no one has sex at the time that you have just delivered”* (AGYW, IDI, married, accepted but later discontinued PrEP).

To address this, providers noted the need for various methods to improve and monitor PrEP adherence. Some providers suggested biomedically monitoring drug levels, while others emphasized the need for patient support: *“Ongoing counselling and continuous stressing on the importance of adherence can help… in as much as people have their own choices, we do routinely counsel them on adherence. Though it still depends on their personal choice, we must still encourage them”* (nurse, FGD). However, while adherence to a daily regimen was commonly discussed among providers, it was not explicitly mentioned among AGYW.

Aside from concerns with PrEP literacy, the stigma surrounding PrEP, and specifically its similarity to ARVs and the implication of HIV stigma, were frequently cited as problematic among AGYW: *“They don’t understand even if you say it is PrEP… They say that [the pills] are ARVs, even though you are not sick”* (AGYW, FGD, married, accepted PrEP). Furthermore, the resemblance between ARVs and PrEP packaging was particularly an issue; one participant described burning the pill bottle after the medication was finished to avoid being misperceived as having HIV: *“When I got PrEP, I decided to burn the bottles once I was done with the first one, and my neighbor saw me and said ‘Ah, so you are our friend.’ I said no; she kept on saying ‘You don’t have to deny it, those bottles [are ARVs]’. I gave her the bottles to have a look and she was embarrassed… she later said ‘Ah please don’t tell others; I thought we are in the same group”* (AGYW, FGD, unmarried, accepted PrEP). Health care providers noted similar misperceptions of PrEP indicating a positive HIV status: *“Especially those that are married and were given PrEP after presenting to the clinic with STIs - once their partner learned that they are taking PrEP, they would think that PrEP are ARVs. This resulted in disruption in their marriages”* (nurse, FGD).

### PrEP access and service delivery: Nuanced preferences among AGYW and a need for holistic counseling

Many AGYW indicated that an HIV clinic was not their preferred location to access PrEP because they feared being seen by people they knew: *“If I meet a church elder here [at the HIV clinic], can I tell him that I am here to see a pregnant woman? Or came for malaria medication? No! I will still have to explain to them why I am here; it is not easy”* (AGYW, FGD, unmarried, accepted PrEP). Although both health facilities in the study offer HIV testing services, participants stated that it was assumed you would be at such a location to access treatment, and therefore had HIV. Additionally, many participants noted difficulties in receiving care in a timely manner. Several participants described reporting to a health facility for a scheduled appointment having to wait several hours to be seen, and subsequently missing school or work: *“They tell us to be reporting to the facility at 8am; we come here at 8 and we get up to 11am without being assisted… One time I got permission from my boss that I was coming here and the time that I was excused passed; my boss told me not to report for work on that day, which can risk losing the job.”* (AGYW, FGD, unmarried, accepted PrEP).

Despite these barriers, many AGYW indicated that hospitals and health facilities (including HIV clinics) are trusted sources for receiving services and accurate information: *“[If] I have a question about PrEp I would ask the clinician or the nurse of the clinic where I access the drugs because I feel like they are flexible and approachable to answer any question”* (AGYW, FGD, unmarried, accepted PrEP). In urban Lilongwe, the health facilities in the study were also noted as being ideal access points, and that not being misperceived as accessing ARVs was important: *“… it would be good for us to continue taking PrEp at the place where we used to get it at the time of the study unlike the hospital where there are a lot of people. It was good during the study because it was just us”* (AGYW, FGD, unmarried, accepted PrEP). These results suggest that for AGYW to feel comfortable accessing PrEP, convenience (both in location and waiting time) are critical, and that HIV stigma remains a barrier in service delivery.

Some AGYW noted difficulties with a daily pill regimen and subsequently declined PrEP: *“I reported to the clinic where they told me to choose whether I will be taking the drugs or not. I find it difficult to be taking medication and I cannot manage; it is better for me to just come and get tested*” (AGYW, FGD, unmarried, declined PrEP). Given this barrier, both AGYW and providers reported interest in injectable PrEP: *“We have been receiving question if we have injectable drugs, where one will be injected once a month. They ask, ‘When will this start?’”* (clinician, FGD); *“I do not take any medication unless there is another way to take the drugs, may be through injection. I would love to take the medication, but I find it difficult to swallow the drugs”* (AGYW, FGD, married, declined PrEP). Participants noted that an injectable method avoids the hassle of a daily pill and also removes the stigma associated with PrEP being mistaken for ARVs: *“That [injectable PrEP] will help because PrEp packaging makes them look like ARVs and people find it difficult to differentiate; they associate us with taking ARVs”* (AGYW, FGD, unmarried, accepted PrEP). Furthermore, many AGYW noted they are already familiar with the process of receiving an injection in a health facility on a regular basis for family planning: *“We should be injected just like it is with family planning methods, where one can be injected once every three months”* (AGYW, FGD, unmarried, accepted PrEP).

The impact of PrEP access on condom use was also noted by both AGYW and health care providers. Some AGYW noted that they were afraid of HIV, but not of STIs or pregnancy, explaining “*Before I started taking PrEP, sometimes I would use condoms but since I started taking PrEP, I have no use for a condom… I wanted the condom to protect me from getting the disease and now PrEP is doing the same purpose. Why should I use it?”* (AGYW, FGD, unmarried, accepted PrEP). Given this disincentive to use condoms, health care providers indicated the need for holistic sexual and reproductive health counseling: *“We also provide counselling that they should use dual protection, but in case it becomes difficult then [PrEP] helps them to be protected”* (nurse, FGD). One health care provider elaborated *“When you tell them that you can still get pregnant and STI even though you are using PrEP... They respond to say “Those are curable, we will come here and will be injected with Gentamicin; I will then be okay, unlike when I get infected with HIV, I will have to live with it for my entire life. If I get infected with syphilis I can come here, you will give me medicine. If I get pregnant, then I will deliver and take care of my child’”* (clinician, FGD).

## Discussion

The results from this study suggest that AGYW with higher risk of HIV acquisition in urban areas of Lilongwe, Malawi had multiple sexual risk factors but were also motivated to protect themselves from HIV and interested in using PrEP as a prevention method. Specifically, our study highlights the importance of transactional sex as a risk factor; AGYW who reported transactional sex in the past six months were more likely to take PrEP than AGYW who did not. Our results align with previous research showing a strong interest in PrEP in Malawi and other low-resource settings [9,15] and high initial uptake among populations most at risk for HIV [11].

Drawing from PMT, we outlined the cognitive mediating process of AGYW in determining the threat appraisal of HIV. AGYW perceived themselves at high risk for HIV, describing several risk factors, including financial instability and inability to negotiate condom usage. These findings are supported by earlier research in the southern Africa region indicating that condom use can be particularly difficult for AGYW to negotiate given norms around power and violence in relationships [6]. Additionally, HIV stigma, and particularly its effect on relationships and life events such as marriage, highlighted the severity of an HIV diagnosis for AGYW. This awareness of HIV risk (perceived vulnerability), fear of HIV acquisition, and limited agency in protecting oneself all informed AGYW’s decisions to participate in the study and take PrEP.

Stigma was described in a multitude of ways and heavily influenced all aspects of the PrEP experience including individual decision making, access and delivery strategies, and relationship dynamics. Thus, it is important to consider the potential high response cost of using PrEP for AGYW. Stigma around PrEP being used to mitigate high risk behaviors (e.g., multiple sexual partners) and similarity to ARVs (implicating HIV stigma) were frequently cited by AGYW. Other studies conducted in the region [33,34] have found similar results, with HIV and PrEP stigma representing significant barriers for uptake and adherence. Given the impact of these intersecting stigmas, social and behavior change communication for AGYW, as well as their parents and sexual partners, may be helpful in PrEP education and demand creation.

Many AGYW participants noted that PrEP’s similarity and proximity to ARVs and associated service delivery points was problematic. This conflation of PrEP use and having HIV, and the resulting stigma, has been documented throughout the region [35,36]. Given that AGYW in our study cited both health facilities and providers as sources of trusted, accurate information, further research to identify optimal locations for PrEP service delivery within a health facility (e.g., pharmacy versus exam room, PrEP-specific pickup point versus integrated with other services, etc.) may be helpful to increase PrEP uptake and adherence among AGYW.

For AGYW’s coping appraisal, participants highlighted the response efficacy, defined as the belief that PrEP is effective at preventing HIV, as a driving factor in changing their behavioral intentions; highlighting this high degree of response efficacy may be a critical component of future PrEP programming for AGYW. Where fear of an HIV diagnosis had previously prevented protective behaviors such as HIV testing, PrEP provided a sense of autonomy and control over HIV. AGYW participants spoke to feeling empowered to use PrEP to protect themselves, often without the knowledge or support of sexual partners. Many participants discussed this self-efficacy within the context of risk factors outside of their locus of control: condom negotiation with an unwilling partner, a partner’s sexual activity outside of their relationship, partner with an unknown HIV status, etc. These contextual factors highlight the importance of AGYW’s self-efficacy in arriving at a protection motivation and their decision to use PrEP, despite the response costs described above. A focus on this self-efficacy may be a critical component of PrEP programming for AGYW, particularly in demand creation.

AGYW’s behavioral intentions around PrEP also highlighted a key difference in their threat appraisal: many AGYW noted a fear of HIV, but not of STIs or pregnancy. This translated to a disincentive to use condoms while taking PrEP, which aligns with existing literature [37]. While health care workers expressed concerns regarding reduced condom use, AGYW themselves did not express similar concerns. This misalignment, along with the impact of dual protection on public health outcomes, highlights the importance of client-centered services, especially for biomedical HIV prevention among AGYW. Support and training for health care workers to provide non-stigmatizing, youth-friendly health services, as well as support from AGYW themselves as peer educators and ambassadors, may be helpful in this regard.

Despite enthusiasm around PrEP as a preventive measure, both providers and AGYW in our study noted misconceptions around PrEP’s effectiveness among AGYW and that it was sometimes only being taken in the days immediately leading up to and following sexual activity As such, PrEP literacy materials may emphasize that daily adherence is required for effectiveness, and such materials will be crucial components of demand creation activities in the community, as well as in PrEP intake and refill visits in health facilities. Future research may benefit from identifying and evaluating strategies to support PrEP adherence and retention in care.

Additionally, the provision of PrEP education and adherence support may expand the scope of practice for some health care providers. Comprehensive training for nurses and clinicians is a critical component of scale up, as well as developing service delivery models which minimize the burden on already over-stretched health systems, such as peer support [38] for AGYW. Training should equip providers to reduce stigma around sexual activity and PrEP use, as well as address common misconceptions to improve uptake and adherence.

AGYW participants also expressed interest in a long-acting, injectable form of PrEP, echoing results from the 2017–2018 Malawi Longitudinal Study of Families and Health, which found that the majority of respondents aged 10–16 years (both male and female) would prefer an injection administered at a health facility over a daily pill as an HIV prevention regimen [16]. Having a differentiated service delivery approach, such as an option between a daily pill regimen or a long-acting injectable, may increase PrEP access and use among AGYW [39]. The regulatory approval of Cabotegravir in Malawi in March 2023 represents significant progress in making effective HIV prevention methods easier to access for AGYW in Malawi [40]. However, for scale up, increasing PrEP knowledge and literacy around its use, particularly for AGYW, is critical. In Malawi, a nationally representative survey found that only 11.8% of females aged 15–19 years had heard of PrEP [3].

### Limitations

Limitations of our study include the possibility of social desirability bias; providers and AGYW may have been unwilling to share some perspectives on sensitive topics such as stigma and sexual risk factors. Additionally, the descriptive statistics presented here are not representative of the larger mixed methods study population. While the use of purposive sampling helped to ensure that a variety of experiences and perceptions were reflected, the perspectives of AGYW with higher risk for HIV acquisition in Lilongwe may not be representative of similar populations across Malawi or southern Africa.

## Conclusion

These data highlight that AGYW are interested in using PrEP to protect themselves from HIV infection. Given complex power dynamics in their relationships, PrEP affords AGYW agency to make choices that ensure their sexual health and well-being. AGYW may benefit from innovative service delivery models that minimize stigma and offer other reproductive health services, such as contraception, for added convenience and comprehensive service access. Additionally, AGYW and their providers would benefit from education about PrEP effectiveness and HIV risk perception to improve use of PrEP. Prioritizing AGYW for PrEP service delivery, including new biomedical prevention strategies, may facilitate HIV epidemic control.

## Supporting information

S1 Checklist Inclusivity in global research questionnaire. (DOCX)
